# Age‐related white matter changes revealed by a whole‐brain fiber‐tracking method in bipolar disorder compared to major depressive disorder and healthy controls

**DOI:** 10.1111/pcn.13166

**Published:** 2020-11-09

**Authors:** Yoshikazu Masuda, Go Okada, Masahiro Takamura, Chiyo Shibasaki, Atsuo Yoshino, Satoshi Yokoyama, Naho Ichikawa, Shiho Okuhata, Tetsuo Kobayashi, Shigeto Yamawaki, Yasumasa Okamoto

**Affiliations:** ^1^ Department of Psychiatry and Neuroscience Hiroshima University Hiroshima Japan; ^2^ Graduate School of Engineering Kyoto University Kyoto Japan

**Keywords:** bipolar disorder, diffusion tensor imaging, major depressive disorder, white matter

## Abstract

**Aim:**

Several studies have reported altered age‐associated changes in white matter integrity in bipolar disorder (BD). However, little is known as to whether these age‐related changes are illness‐specific. We assessed disease‐specific effects by controlling for age and investigated age‐associated changes and Group × Age interactions in white matter integrity among major depressive disorder (MDD) patients, BD patients, and healthy controls.

**Methods:**

Healthy controls (*n* = 96; age range, 20–77 years), MDD patients (*n* = 101; age range, 25–78 years), and BD patients (*n* = 58; age range, 22–76 years) participated in this study. Fractional anisotropy (FA) derived from diffusion tensor imaging in 54 white matter tracts were compared after controlling for the linear and quadratic effect of age using a generalized linear model. Age‐related effects and Age × Group interactions were also assessed in the model.

**Results:**

The main effect of group was significant in the left column and body of the fornix after controlling for both linear and quadratic effects of age, and in the left body of the corpus callosum after controlling for the quadratic effect of age. BD patients exhibited significantly lower FA relative to other groups. There was no Age × Group interaction in the tracts.

**Conclusion:**

Significant FA reductions were found in BD patients after controlling for age, indicating that abnormal white matter integrity in BD may occur at a younger age rather than developing progressively with age.

Diffusion tensor imaging (DTI) is a technique that enables the evaluation of white matter microstructures by quantifying the extension and direction of water molecule diffusivity.^1^ An increasing number of studies have applied DTI to investigate major depressive disorder (MDD) and bipolar disorder (BD). Abnormal white matter integrity has been shown to play an essential role in the pathophysiology of MDD^2^, ^3^, ^4^, ^5^, ^6^ and BD.^7^, ^8^, ^9^ However, common or disease‐specific mechanisms of white matter degeneration among the two disorders remain to be elucidated. Several DTI studies have directly compared white matter integrity between the two disorders to identify disease‐specific abnormalities.^10^, ^11^, ^12^, ^13^, ^14^ However, the results have been inconsistent because of: (i) mismatched participant characteristics, such as age, sex, and disease severity; (ii) different methods of DTI data analysis; and (iii) insufficient statistical power. The ENIGMA‐DTI workgroup has reported a large meta‐analysis comparing white matter deficit patterns among schizophrenia, BD, MDD, obsessive–compulsive disorder, post‐traumatic stress disorder, and traumatic brain injury patients. The patients with BD and MDD showed similar negative effect size on average FA values, and disease‐specific, significant regional reduction patterns in FA values.^15^

It is known that alterations of white matter integrity occur with age across the life span. The whole brain fractional anisotropy (FA) of healthy individuals shows a quadratic trajectory with an age peak in the fourth decade of life,^16^ and regional variations in the rate and the timing of neural maturation of white matter.^17^, ^18^, ^19^, ^20^ In contrast, age‐related white matter changes in individuals with psychiatric diseases seem to deviate from the typical trajectory. For instance, an accelerated rate of white matter deterioration with age was demonstrated in schizophrenia.^21^, ^22^, ^23^ Multiple and complex factors, such as risk genes, disease neurotoxicity, excessive aging effects, and medication, might contribute to the abnormal trajectory of white matter.^24^ MDD and BD are chronic recurring disorders, and genetic contributions are strongly suspected in their etiology. Therefore, it was postulated that an atypical trajectory of white matter would be observed in these two mood disorders. Indeed, the ENIGMA MDD working group examined age‐related changes of white matter in MDD using the largest sample available to date and reported Group × Age interactions in the genu of the corpus callosum, the body of the corpus callosum, the fornix/stria terminalis, and the sagittal stratum.^25^ Moreover, cross‐sectional studies examining correlations between white matter integrity and age in BD patients show an aberrant neurodevelopmental process in the corpus callosum and inferior longitudinal fasciculus.^26^ Moreover, an accelerated rate of white matter deterioration with age in the uncinate fasciculus, the hippocampal portion of the cingulum, and the genu and splenium of the corpus callosum have been reported.^27^, ^28^ Investigating age‐related changes is essential for understanding the common or disease‐specific mechanisms of white matter degeneration in the two diseases. However, to the best of the authors' knowledge, only a few studies have directly compared age‐related changes in white matter integrity between BD and MDD.

In this study, we directly compared age‐associated changes in white matter integrity between the two disorders to clarify illness‐specific mechanisms of white matter degeneration. We also examined the correlation between the duration of illness and white matter integrity using the identical method to clarify whether disease neurotoxicity is associated with white matter changes in the two disorders. A generalized linear model was performed to compare FA after controlling for age and the duration of illness among BD patients, MDD patients, and healthy controls (HC). We report the results of FA values of whole‐brain average and 54 regional white matter tracts. The FA values in each white matter tract were calculated using automated whole brain atlas‐based tractography with the method described by Okuhata *et al*.^29^

## Methods

### Participants

Patients with MDD (*n* = 101; age range, 25–78 years) and BD (*n* = 58; age range, 22–76 years) were included in the study. All the patients were outpatients at the Hiroshima University Hospital psychiatry or medical research institutions in Hiroshima city. HC (*n* = 98; age range, 20–77 years) with no previous psychiatric history were solicited through a newspaper advertisement. An expert clinician had diagnosed the patients according to the DSM‐5. The Mini‐International Neuropsychiatric Interview (MINI) was performed at the time of participation in the study to confirm the diagnosis. We also conducted the MINI on HC to verify that the HC participants did not meet the criteria for any psychiatric disorder. All participants were right‐handed and native speakers of Japanese. Exclusion criteria for the study were: (i) comorbid diagnosis of schizophrenia, alcohol or substance abuse/dependence, dementia, developmental disorders, eating disorders, or personality disorder; (ii) severe physical illness; (iii) high risk for suicide; and (iv) currently breastfeeding, pregnancy, or post‐partum period. Self‐assessments of symptom severity were conducted by all the participants using the Beck Depression Inventory‐II (BDI‐II) and the Altman Self‐Rating Mania Scale (ASRM). Structured interview assessments of symptom severity in the patient groups were conducted on the day of the MRI scan using the 17‐item Hamilton Rating Scale for Depression (HRSD) and the Young Mania Rating Scale (YMRS). Also, premorbid IQs were estimated using the Japanese Adult Reading Test‐25. All the patients were on medication at the time of MRI scanning. The medications included lithium, antidepressants, anticonvulsants, antipsychotics, and benzodiazepines. We used the strategy described earlier to measure the total medication load.^30^ This study was approved by the Ethics Committee of the Medical Faculty of Hiroshima University, and it was conducted according to the Helsinki Declaration of 1975. All the participants gave their written informed consent before participating in the study. The participants received financial compensation for their assistance.

### MRI data acquisition

All the participants were scanned at Hiroshima University, Hiroshima, Japan, by using a 3.0T Siemens Magnetom (Siemens, Munich, Germany) with a 12‐channel head coil. Diffusion tensor imaging data points were acquired with sequence parameters as follows: 60 slices, repetition time/echo time = 8100 ms/94 ms, field of view = 240 mm, voxel size = 2.5 × 2.5 × 2.5 mm^3^, image matrix = 96 × 96, 30 non‐collinear directions of motion probing gradient. The b value was b = 1000 s/mm^2^, and one image was also acquired with b = 0.

### Data preprocessing and fiber tracking

Data preprocessing and atlas‐based whole brain fiber tracking was performed according to the method described by Okuhata *et al*.^29^ Preprocessing was done by using the Functional MRI of the Brain (FMRIB) Software Library. First, non‐brain tissue was deleted with the brain extraction tool from eddy‐current‐corrected diffusion MRI data. Then, diffusion indices, such as FA, tensors, and the first eigenvector, were calculated using the FMRIB Diffusion Toolbox. Finally, linear and non‐linear registrations were conducted using the FMRIB Linear Image Registration Tool followed by the FMRIB Nonlinear Image Registration Tool. The automated fiber tracking with tensor deflection method was performed with the 54 white matter parcels, which were prescribed based on the Johns Hopkins University Diffusion Tensor Imaging‐based white‐matter atlas. The FA value at each stepping point (stepping width: 0.5 mm) along each fiber was calculated by interpolation using the volume data for the center points of the nearest eight voxels around the stepping point. The terminate criteria were: FA < 0.25 and flip angle >45°. Fiber tracking procedures were performed using matlab for Windows (Ver. R2015b; MathWorks, Natick, MA, USA).

### Statistical analysis

Differences in age, estimated IQ, BDI‐II score, and ASRM score among the three groups were assessed using one‐way analyses of variance models. The χ^2^‐test assessed differences in sex distribution among the three groups. Independent‐sample *t*‐tests assessed differences in illness duration, number of depressive episodes, the HRSD score, and the YMRS score. A significance level of *P* < 0.05 was set for demographic and clinical characteristics, and statistical analyses were conducted using IBM spss Version 24 for Windows (SPSS Japan, Tokyo, Japan). The effects of age, group, and Age × Group interactions on the average FA and FA in the 54 white matter tracts were modeled using a generalized linear model to examine age‐related FA changes. Sex, mood states, and the medication load index were entered into the model as covariates. Based on a statistical approach previously reported by Kochunov *et al*., we performed two types of modeling: (i) including the linear effect of age; and (ii) including the quadratic effect of age.^23^

The model structure is described by:

$$\begin{array}{c} {\mathrm{FA}}_{i,j}\sim C+\beta_{1}{\mathrm{Age}}_{i}+\beta_{2}{\mathrm{dx}}_{j}+\beta_{3}{\mathrm{Age}}_{i}∙{\mathrm{dx}}_{j}+{\mathrm{cov}}_{i,j}\\ i=\left(1,2∙∙∙,n\right)\\ j=\left[\begin{array}{c} \;\left(1,0,0\right)=\text{major depressive disorder}\\ \left(0,1,0\right)=\text{bipolar disorder}\;\\ \left(0,0,1\right)=\text{healthy control}\; \end{array}\right], \end{array}$$

where C is the constant FA term, *β* is the covariate regression coefficient for each covariate, Age_i_ is the main effect of age for the i^th^ individual, *dx*_*j*_ is the main effect for group, and cov is the coefficient that accounts for covariates. We also performed modeling to examine the linear effect of duration of illness on the average FA and FA in the 54 white matter tracts for the patients with MDD and BD.

The model structure is described by:

$$\begin{array}{c} {FA}_{i,j}\sim C+\beta_{1}{\text{Duration}}_{i}+\beta_{2}{\mathrm{dx}}_{j}+\beta_{3}{\text{Duration}}_{i}∙{\mathrm{dx}}_{j}+{\mathrm{cov}}_{i,j}\\ i=\left(1,2∙∙∙,n\right)\\ j=\left[\begin{array}{c} \;0=\text{major depressive disorder}\\ 1=\text{bipolar disorder}\; \end{array}\right], \end{array}$$

where *Duration*_*i*_ is the main effect of duration of illness for the i^th^ individual.

A significant level of *P* < 0.001 was selected for these models to correct for multiple comparisons (*n* = 55). (The threshold level of significance shown in the table is *P* < 0.05 uncorrected. However, the discussion and conclusions are limited to results that exceed a significance level of *P* < 0.001). Modeling was conducted with R Package Version 3.4.3 (https://www.r-project.org/).

Bonferroni's post‐hoc analysis was conducted with the significance level set at *P* < 0.05 to estimate differences in the mean FA of white matter tracts between the three groups with a main effect of group.

## Results

### Demographic and clinical characteristics

Summaries of the demographic and clinical characteristics are shown in Table 1. At the time of the MRI scanning, 40 MDD patients and 32 BD patients were in a state of euthymia (HRSD < 8, YMRS < 8), whereas 61 MDD patients and 26 BD patients were in a state of depression (HRSD ≥ 8, YMRS < 8). There was no significant difference in age, sex distribution, estimated IQ, or ASRM among the three groups. A significant main effect of group was detected in BDI‐II score across the three groups (MDD = 17.7, BD = 16.1, HC = 8.7, *F* = 21.886, *P* < 0.001). Post‐hoc group comparisons indicated that BDI‐II scores were significantly higher in the patient groups compared to the HC group. The HRSD score was significantly higher in MDD patients than BD patients (MDD = 10.6, BD = 6.5, *t* = 3.424, *P* < 0.01), and the YMRS score was significantly higher in BD patients than MDD patients (MDD = 0.7, BD = 2.1, *t* = 3.429, *P* < 0.01). The BD patients had a significantly longer duration of illness (MDD = 7.6 years, BD = 17.4 years, *t* = 6.675, *P* < 0.001), and had a greater number of depressive episodes (MDD = 3.2, BD = 10.8, *t* = 7.540, *P* < 0.001) than MDD patients. The medication load index was significantly higher in BD patients than MDD patients (MDD = 2.3, BD = 3.0, *t* = 3.732, *P* < 0.01; see Table 1 for details).

**Table 1:** Demographics, clinical characteristics, and psychiatric medication for study participants

	MDD (*n* = 101)	BD (*n* = 58)	HC (*n* = 98)	Statistics	*P*	Post‐hoc
Age (years)	50.5 ± 13.2	52.0 ± 12.5	53.7 ± 13.2	*F* = 1.507	0.223	
Sex (male/female, *n*)	41/60	29/29	35/63	χ^2^ = 3.082	0.214	
BDI‐II	17.7 ± 11.7	16.1 ± 12.0	8.7 ± 6.3	*F* = 21.886	<0.001	BD, MDD > HC
ASRM	1.5 ± 2.2	1.6 ± 1.8	0.9 ± 2.0	*F* = 2.696	0.07	
HRSD	10.6 ± 8.3	6.5 ± 5.2	—	*t* = 3.424	<0.01	
YMRS	0.7 ± 1.4	2.1 ± 2.9	—	*t* = 3.429	<0.01	
Duration of illness (years)	7.6 ± 7.3	17.4 ± 11.1	—	*t* = 6.675	<0.001	
Number of depressive episodes	3.2 ± 4.3	10.8 ± 8.4	—	*t* = 7.540	<0.001	
Estimated IQ	105.7 ± 10.6	105.7 ± 9.8	106.5 ± 7.9	*F* = 0.245	0.783	
Medication load index	2.3 ± 1.1	3.0 ± 1.1	—	*t* = 3.732	<0.001	
Antidepressant, % (*n*)	94.1 (95)	34.5 (20)	—	χ^2^ = 65.335	<0.001	
SSRI	44.6 (45)	5.2 (3)	—	χ^2^ = 27.113	<0.001	
SNRI	28.7 (29)	13.8 (8)	—	χ^2^ = 4.593	<0.05	
NaSSA	17.8 (18)	3.4 (2)	—	χ^2^ = 6.922	<0.01	
Tricyclic antidepressants	8.9 (9)	0	—	χ^2^ = 5.478	<0.05	
Others	7.9 (8)	12.1 (7)	—	χ^2^ = 0.742	0.389	
Lithium, % (*n*)	8.9 (9)	65.5 (38)	—	χ^2^ = 56.697	<0.001	
Anticonvulsant, % (*n*)	0 (0)	58.6 (34)	—	χ^2^ = 75.311	<0.001	
Antipsychotic, % (*n*)	16.8 (17)	46.5 (27)	—	χ^2^ = 16.259	<0.001	
Benzodiazepine, % (*n*)	78.2 (79)	62.0 (36)	—	χ^2^ = 4.800	<0.05	

ASRM, Altman Self‐Rating Mania Scale; BD, bipolar disorder; BDI‐II, Beck Depression Inventory‐II; HC, healthy controls; HRSD, Hamilton Rating Scale for Depression; MDD, major depressive disorder; NaSSA, noradrenergic and specific serotonergic antidepressant; YMRS, Young Mania Rating Scale.

### Effects of age, group, and Age × Group interaction on the average FA and FA in the 54 tracts of the three groups

Beta coefficients and significance of the main effects of age, group, and Age × Group interaction on the FA in the tracts in which significant results (*P* < 0.05) were obtained are summarized in Table 2 (full results for all tracts are shown in Table S1). There was a significant main effect of age in the average FA and number of white matter tracts, including in the corpus callosum. The main effect of group was significant only for the left column and the body of the fornix. There was no significant Age × Group interaction effect.

**Table 2:** Beta coefficients and significance for effect of age, group, and Age × Group interaction for the FA values in white matter tracts in which significant results (*P* < 0.05) were obtained based on linear age effect model for MDD patients, BD patients, and HC

	Intercept	Main effect of age β	Group	Main effect of group β	Age × Group interaction β
Average FA	0.48 ± 0.01	−7.16 ± 1.03•10–4***	MDD	−2.71 ± 8.75•10–3	7.59 ± 15.19•10–5
			BD	−3.12 ± 0.96•10–2**	3.73 ± 1.75•10 − 4*
Inferior cerebellar peduncle R	0.45 ± 0.01	−2.24 ± 2.26•10–4	MDD	−4.44 ± 2.04•10–2	7.57 ± 3.37•10–4*
			BD	−3.87 ± 2.20•10–2	5.14 ± 3.86•10–4
Superior cerebellar peduncle L	0.48 ± 0.01	4.28 ± 1.51•10–4**	MDD	4.01 ± 13.67•10–3	4.43 ± 22.53•10–5
			BD	−1.68 ± 1.47•10–2	2.43 ± 2.58•10–4
Superior cerebellar peduncle R	0.54 ± 0.01	3.72 ± 1.56•10–4*	MDD	−2.07 ± 1.40•10–2	3.99 ± 2.31•10–4
			BD	−2.43 ± 1.51•10–2	2.99 ± 2.64•10–4
Cerebral peduncle L	0.55 ± 0.01	−4.88 ± 1.73•10–4**	MDD	−4.24 ± 15.56•10–4	9.46 ± 25.63•10–5
			BD	−6.54 ± 167.5•10–4	−1.49 ± 2.94•10–4
Cerebral peduncle R	0.61 ± 0.01	**−9.63 ± 1.90•10–4** ***	MDD	−2.36 ± 1.71•10–2	5.79 ± 2.84•10–4*
			BD	−1.62 ± 1.85•10–2	3.29 ± 3.24•10–4
Anterior limb of internal capsule L	0.60 ± 0.01	**−9.71 ± 2.11•10–4** ***	MDD	4.38 ± 19.04•10–3	−6.38 ± 31.38•10–5
			BD	−2.15 ± 2.05•10–2	1.46 ± 3.60•10–4
Anterior limb of internal capsule R	0.59 ± 0.01	**−8.18 ± 1.99•10–4** ***	MDD	−1.27 ± 17.97•10–3	4.88 ± 296.2•10–6
			BD	−4.51 ± 1.94•10–2	6.63 ± 3.40•10–4
Posterior limb of internal capsule L	0.58 ± 0.01	−3.51 ± 1.70•10–4*	MDD	1.47 ± 1.53•10–2	−3.23 ± 2.53•10–4
			BD	−2.40 ± 1.65•10–2	3.36 ± 2.90•10–4
Posterior thalamic radiation L	0.49 ± 0.01	**−1.10 ± 0.21•10–3** ***	MDD	−6.35 ± 1.85•10–2	1.24 ± 3.04•10–4
			BD	−2.15 ± 1.98•10–2	2.21 ± 3.49•10–4
Posterior thalamic radiation R	0.53 ± 0.02	**−1.51 ± 0.27•10–3** ***	MDD	−4.81 ± 23.98•10–3	3.22 ± 3.95•10–4
			BD	−4.93 ± 2.58•10–2*	7.14 ± 4.53•10 − 4
Anterior corona radiata L	0.42 ± 0.01	**−1.17 ± 0.17•10–3** ***	MDD	−6.40 ± 16.96•10–3	9.09 ± 26.31•10–5
			BD	−2.51 ± 2.57•10–2	2.55 ± 3.02•10–4
Anterior corona radiata R	0.40 ± 0.01	**−1.13 ± 0.16•10–3** ***	MDD	3.12 ± 15.52•10–3	5.11 ± 25.58•10–5
			BD	−3.61 ± 1.67•10–2	6.15 ± 2.94•10–4
Superior corona radiata L	0.43 ± 0.01	−4.78 ± 1.78•10–4**	MDD	8.40 ± 16.03•10–3	−1.13 ± 2.64•10–4
	BD	−3.47 ± 1.73•10–2	5.30 ± 3.03•10–4		
Superior corona radiata R	0.45 ± 0.01	−4.54 ± 1.73•10–4**	MDD	1.77 ± 1.56•10–2	−3.18 ± 2.56•10–4
			BD	−3.76 ± 1.68•10–2	3.97 ± 2.94•10–4
Posterior corona radiata R	0.43 ± 0.01	−5.51 ± 2.21•10–6*	MDD	6.38 ± 19.94•10–3	2.22 ± 32.87•10–5
			BD	−3.18 ± 2.15•10–2	4.65 ± 3.77•10–4
Cingulum (cingulate gyrus) L	0.46 ± 0.01	−6.88 ± 2.08•10–4**	MDD	−1.68 ± 18.75•10–3	9.70 ± 30.91•10–5
			BD	−6.09 ± 2.02•10–2**	7.17 ± 3.54•10 − 4*
Cingulum (cingulate gyrus) R	0.48 ± 0.02	**−9.26 ± 2.62•10–4** ***	MDD	3.08 ± 2.36•10–2	−4.31 ± 3.89•10–6
			BD	−5.34 ± 2.54•10–2*	7.28 ± 4.46•10 − 4
Cingulum (hippocampus) L	0.39 ± 0.01	−7.96 ± 2.34•10–4**	MDD	−1.10 ± 2.10•10–2	2.86 ± 3.46•10–4
			BD	−3.34 ± 2.27•10–2	3.60 ± 3.97•10–4
Cingulum (hippocampus) R	0.42 ± 0.01	**−1.18 ± 0.23•10–3** ***	MDD	−1.50 ± 2.08•10–2	4.67 ± 3.43•10–4
			BD	−6.86 ± 2.24•10–2**	1.17 ± 0.39•10 − 3**
Fornix (cres) L	0.45 ± 0.01	**−1.32 ± 0.19•10–3** ***	MDD	−1.18 ± 1.74•10–2	1.91 ± 2.87•10–4
			BD	−2.98 ± 1.88•10–2	4.32 ± 3.29•10–4
Fornix (cres) R	0.49 ± 0.01	**−1.52 ± 0.23•10–3** ***	MDD	5.26 ± 20.93•10–3	3.84 ± 34.50•10–5
			BD	−4.75 ± 2.26•10–2	7.21 ± 3.95•10–4
Superior fronto‐occipital fasciculus L	0.41 ± 0.02	**−9.27 ± 2.46•10–4** ***	MDD	1.11 ± 2.21•10–2	−6.38 ± 36.52•10–5
			BD	−3.11 ± 23.87•10–3	9.85 ± 41.86•10–5
Superior fronto‐occipital fasciculus R	0.43 ± 0.01	**−1.23 ± 0.24•10–3** ***	MDD	2.99 ± 21.65•10–3	−2.78 ± 35.69•10–5
			BD	−3.52 ± 2.33•10–2	6.43 ± 4.09•10–4
Inferior fronto‐occipital fasciculus L	0.43 ± 0.01	−1.92 ± 2.07•10–6	MDD	1.10 ± 1.86•10–2	−3.79 ± 3.06•10–4
			BD	3.03 ± 2.00•10–2	−9.46 ± 3.51•10–4**
Sagittal stratum L	0.46 ± 0.01	**−8.48 ± 1.95•10–4** ***	MDD	2.51 ± 17.57•10–3	1.28 ± 2.90•10–4
			BD	−9.99 ± 18.92•10–3	2.87 ± 3.32•10–4
Sagittal stratum R	0.47 ± 0.01	**−1.10 ± 0.23•10–3** ***	MDD	7.09 ± 20.44•10–3	4.31 ± 33.69•10–5
			BD	−1.83 ± 2.12•10–2	4.12 ± 3.87•10–4
External capsule L	0.31 ± 0.01	−5.10 ± 1.55•10–4**	MDD	1.22 ± 1.40•10–2	−1.72 ± 2.31•10–4
			BD	4.75 ± 15.07•10–3	−2.22 ± 2.64•10–4
External capsule R	0.36 ± 0.01	**−5.87 ± 1.69•10–4** ***	MDD	−1.98 ± 15.24•10–3	−1.35 ± 2.51•10–4
			BD	−5.34 ± 16.42•10–3	−1.56 ± 2.88•10–4
Middle cerebellar peduncle L	0.50 ± 0.01	−3.37 ± 1.40•10–6*	MDD	−6.96 ± 12.64•10–3	2.08 ± 2.08•10–4
			BD	−1.30 ± 1.36•10–2	1.39 ± 2.39•10–4
Middle cerebellar peduncle R	0.52 ± 0.01	−3.22 ± 1.36•10–4*	MDD	−3.06 ± 12.20•10–3	−4.31 ± 20.11•10–5
			BD	−8.30 ± 13.14•10–3	−2.02 ± 23.05•10–5
Fornix (column and body of fornix) L	0.33 ± 0.02	**−3.12 ± 0.39•10–3** ***	MDD	−5.66 ± 3.50•10–2	1.16 ± 0.77•10–3*
			BD	**−1.31 ± 3.77•10–2** ***	1.79 ± 66.16•10 − 3**
Fornix (column and body of fornix) R	0.41 ± 0.04	**−3.57 ± 0.57•10–3** ***	MDD	−1.49 ± 51.26•10–3	4.85 ± 8.45•10–4
			BD	−9.94 ± 5.52•10–2	1.01 ± 0.97•10–3
Genu of corpus callosum L	0.68 ± 0.02	**−1.81 ± 0.28•10–3** ***	MDD	−1.09 ± 2.48•10–2	2.32 ± 4.09•10–4
			BD	−4.45 ± 2.68•10–2	3.50 ± 4.69•10–4
Genu of corpus callosum R	0.69 ± 0.02	**−1.80 ± 0.30•10–3** ***	MDD	−4.36 ± 26.72•10–3	1.89 ± 4.40•10–4
			BD	−5.25 ± 2.87•10–2	5.03 ± 5.05•10–4
Body of corpus callosum L	0.63 ± 0.02	**−2.14 ± 0.31•10–3** ***	MDD	−3.15 ± 2.82•10–2	5.94 ± 4.64•10–4
			BD	−8.90 ± 3.04•10–2**	8.80 ± 5.32•10 − 4
Body of corpus callosum R	0.58 ± 0.02	**−1.71 ± 0.35•10–3** ***	MDD	−2.70 ± 3.11•10–2	4.76 ± 5.13•10–4
			BD	−9.03 ± 3.35•10–2**	7.93 ± 5.88•10 − 4
Splenium of corpus callosum L	0.65 ± 0.01	−3.66 ± 2.01•10–4	MDD	1.52 ± 1.80•10–2	−1.78 ± 2.97•10–4
			BD	−3.18 ± 1.94•10–2	4.62 ± 3.41•10–4
Splenium of corpus callosum R	0.72 ± 0.02	**−1.01 ± 0.25•10–3** ***	MDD	−2.87 ± 22.80•10–3	2.16 ± 3.76•10–4
			BD	−5.50 ± 2.46•10–2*	8.81 ± 4.31•10 − 4*
Retrolenticular part of internal capsule L	0.44 ± 0.01	−3.73 ± 1.36•10–4**	MDD	2.03 ± 1.23•10–2	−1.64 ± 2.02•10–4
			BD	−6.66 ± 13.23•10–3	1.87 ± 2.32•10–4
Retrolenticular part of internal capsule R	0.45 ± 0.01	−4.44 ± 1.69•10–4**	MDD	5.66 ± 15.22•10–3	−3.19 ± 25.08•10–5
			BD	−1.70 ± 1.64•10–2	1.85 ± 2.88•10–4

* *P* < 0.05.

** *P* < 0.01.

*** *P* < 0.001.

Significant *P*‐values are set in bold letters after multiple comparison correction, which required *P* < 0.001.

BD, bipolar disorder; FA, fractional anisotropy; HC, healthy controls; L, left; MDD, major depressive disorder; R, right.

### Effects of age^2^, group, and Age^2^ × Group interaction on the average FA and FA in the 54 tracts of the three groups

**Fig. 1:**
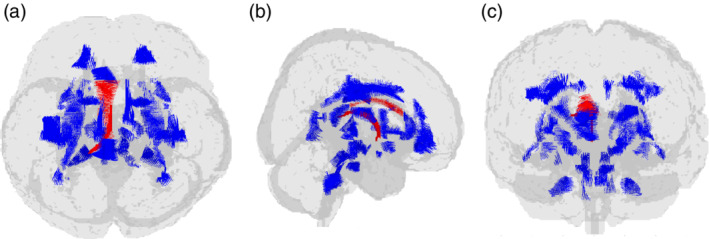
Glass brain view of fractional anisotropy (FA) signal difference among bipolar disorder (BD) patients, major depressive disorder (MDD) patients, and healthy controls (HC), depicted in red. Left image (a) is observed from above, medial image (b) is observed from the right and right image (c) is observed from behind. BD patients show significantly lower mean FA values in the left column and body of fornix, and left body of corpus callosum than MDD patients or HC.

**Fig. 2:**
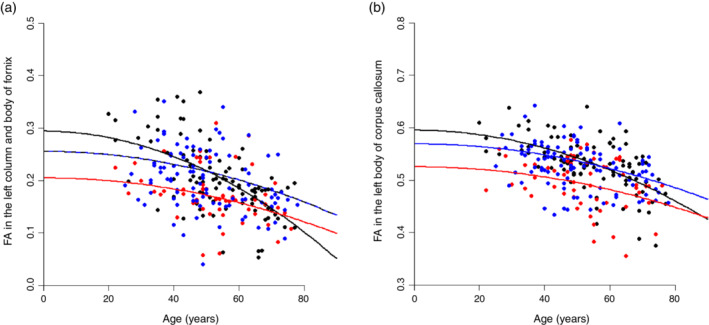
Plots show the age‐related changes of fractional anisotropy (FA) in the (a) left column and body of fornix and (b) left body of corpus callosum. The blue, red, and black lines indicate results of quadratic regression models for each respective group. Significant main effects of group were present in white matter tracts for bipolar disorder (BD) patients. (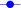) Major depressive disorder patients. (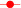) BD patients. (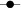) Healthy controls.

Beta coefficients and significance for main effects of age^2^, group, and Age^2^ × Group interaction on the FA in the tracts in which significant results (*P* < 0.05) were obtained are summarized in Table 3 (full results for all tracts are shown in Table S2). There was a significant main effect of age^2^ in the average FA and several white matter tracts similar to the linear effect of age. The main effect of group was significant for the left column and body of the fornix and the left body of the corpus callosum (Figs 1, 2). There was no significant Age^2^ × Group interaction effect.

**Table 3:** Beta coefficients and significance for effect of age^2^, group, and Age^2^ × Group interaction for the FA values in white matter tracts in which significant results (*P* < 0.05) were obtained based on quadratic age effect model for MDD patients, BD patients, and HC

	Intercept	Main effect of age^2^ β	Group	Main effect of group β	Age^2^ × Group interaction β
Average FA	0.47 ± 0.01	−6.82 ± 1.00•10–6***	MDD	−1.39 ± 5.96•10–3	7.83 ± 14.63•10–7
			BD	**−2.17 ± 0.64•10–2** **	3.34 ± 1.70•10 − 6
Inferior cerebellar peduncle L	0.40 ± 0.01	−1.39 ± 2.30•10–6	MDD	−2.80 ± 1.38•10–2*	5.54 ± 3.39•10 − 6
			BD	−3.01 ± 1.49•10–2*	3.83 ± 3.94•10 − 6
Inferior cerebellar peduncle R	0.45 ± 0.01	−2.25 ± 2.10•10–6	MDD	−2.59 ± 1.31•10–2	7.21 ± 3.23•10–6*
			BD	−2.62 ± 1.42•10–2	4.78 ± 3.75•10–6
Medial lemniscus R	0.52 ± 0.01	−6.75 ± 19.77•10–6	MDD	−1.17 ± 1.18•10–2	2.34 ± 28.98•10–7
		BD	−2.89 ± 1.28•10–2*	1.86 ± 3.37•10 − 6	
Superior cerebellar peduncle L	0.46 ± 0.01	4.43 ± 1.46•10–6**	MDD	6.78 ± 8.79•10–3	−2.09 ± 21.57•10–7
			BD	−1.13 ± 0.95•10–2	2.30 ± 2.51•10–6
Superior cerebellar peduncle R	0.55 ± 0.01	3.69 ± 1.50•10–6*	MDD	−9.72 ± 9.04•10–3	3.33 ± 2.22•10–6
			BD	−1.79 ± 0.98•10–2	2.88 ± 2.58•10–6
Cerebral peduncle L	0.54 ± 0.01	−4.49 ± 1.67•10–6**	MDD	2.10 ± 10.07•10–3	9.17 ± 24.71•10–6
			BD	−4.42 ± 10.88•10–3	−9.55 ± 28.73•10–7
Cerebral peduncle R	0.59 ± 0.01	−8.92 ± 1.83•10–6***	MDD	−7.44 ± 11.08•10–3	5.05 ± 2.72•10–6
			BD	−6.98 ± 11.97•10–3	3.02 ± 3.16•10–6
Anterior limb of internal capsule L	0.57 ± 0.01	**−9.21 ± 2.03•10–6** ***	MDD	−2.31 ± 122.5•10–4	−7.02 ± 30.07•10–7
			BD	−1.50 ± 1.32•10–2	8.06 ± 34.96•10–7
Anterior limb of internal capsule R	0.57 ± 0.01	**−7.95 ± 1.91•10–6** ***	MDD	−3.15 ± 1115.4•10–4	−1.73 ± 28.31•10–7
			BD	−2.86 ± 1.25•10–2	6.44 ± 3.29•10–6
Posterior limb of internal capsule L	0.58 ± 0.01	−3.69 ± 1.63•10–6**	MDD	5.98 ± 9.85•10–3	−2.79 ± 2.42•10–6
			BD	−1.58 ± 1.06•10–2	3.31 ± 2.81•10–6
Posterior thalamic radiation L	0.47 ± 0.01	**−1.04 ± 0.20•10–5** ***	MDD	−4.33 ± 11.97•10–3	1.61 ± 2.94•10–6
			BD	−1.45 ± 1.29•10–2	1.92 ± 3.41•10–6
Posterior thalamic radiation R	0.49 ± 0.01	**−1.45 ± 0.25•10–5** ***	MDD	1.93 ± 15.48•10–3	3.59 ± 3.80•10–6
			BD	−3.03 ± 1.67•10–2	6.64 ± 4.42•10–6
Anterior corona radiata L	0.41 ± 0.01	−1.15 ± 0.17•10–5	MDD	−4.36 ± 10.23•10–3	1.15 ± 2.51•10–6
			BD	−1.78 ± 1.11•10–2	2.49 ± 2.92•10–6
Anterior corona radiata R	0.40 ± 0.01	**−1.13 ± 0.16•10–5** ***	MDD	4.13 ± 9.97•10–3	7.22 ± 24.46•10–7
			BD	−1.96 ± 1.08•10–2	5.71 ± 2.85•10–6*
Superior corona radiata L	0.42 ± 0.01	−4.44 ± 1.71•10–6*	MDD	6.19 ± 10.33•10–3	−1.21 ± 2.53•10–6
			BD	−1.98 ± 1.12•10–2	4.50 ± 2.95•10–6
Superior corona radiata R	0.44 ± 0.01	−3.95 ± 1.67•10–6*	MDD	1.10 ± 1.01•10–2	−3.35 ± 2.46•10–6
			BD	−2.63 ± 1.09•10–2	3.41 ± 2.87•10–6
Posterior corona radiata R	0.42 ± 0.01	−5.18 ± 2.14•10–6*	MDD	7.63 ± 12.85•10–3	4.48 ± 31.53•10–7
			BD	−2.04 ± 1.39•10–2	4.58 ± 3.66•10–6
Cingulum (cingulate gyrus) L	0.45 ± 0.01	−6.50 ± 2.02•10–6**	MDD	7.56 ± 120.9•10–4	9.36 ± 29.67•10–7
			BD	−4.26 ± 1.31•10–2	6.60 ± 3.45•10–6
Cingulum (cingulate gyrus) R	0.46 ± 0.01	**−8.90 ± 2.52•10–6** ***	MDD	2.02 ± 1.52•10–2	−3.99 ± 3.73•10–6
			BD	−3.48 ± 1.64•10–2*	6.91 ± 4.33•10 − 6
Cingulum (hippocampus) L	0.37 ± 0.01	**−7.55 ± 2.26•10–6** ***	MDD	−4.94 ± 13.57•10–3	3.07 ± 3.33•10–6
			BD	−2.32 ± 1.47•10–2	3.10 ± 3.87•10–6
Cingulum (hippocampus) R	0.39 ± 0.01	**−1.13 ± 0.22•10–5** ***	MDD	−3.49 ± 13.41•10–3	4.49 ± 3.29•10–6
			BD	−3.75 ± 1.45•10–2**	1.05 ± 0.38•10 − 5**
Fornix (cres) L	0.42 ± 0.01	**−1.28 ± 0.18•10–5** ***	MDD	−7.38 ± 11.20•10–3	2.10 ± 2.75•10–6
			BD	−1.63 ± 1.21•10–2	3.55 ± 3.19•10–6
Fornix (cres) R	0.46 ± 0.01	**−1.45 ± 0.22•10–5** ***	MDD	5.33 ± 13.49•10–3	8.11 ± 33.10•10–7
			BD	−2.60 ± 1.46•10–2	5.98 ± 3.85•10–6
Superior fronto‐occipital fasciculus L	0.38 ± 0.01	**−8.38 ± 2.39•10–6** ***	MDD	9.99 ± 14.33•10–3	−6.58 ± 35.16•10–7
			BD	3.75 ± 15.48•10–3	−2.18 ± 40.88•10–7
Superior fronto‐occipital fasciculus R	0.40 ± 0.01	**−1.17 ± 0.23•10–5** ***	MDD	2.30 ± 13.98•10–3	−1.25 ± 34.29•10–7
			BD	−1.78 ± 1.51•10–2	5.92 ± 3.98•10–6
Inferior fronto‐occipital fasciculus L	0.42 ± 0.01	−1.61 ± 2.00•10–6	MDD	2.99 ± 11.98•10–3	−3.84 ± 2.94•10–6
			BD	9.90 ± 12.94•10–3	−9.53 ± 3.42•10–6**
Sagittal stratum L	0.44 ± 0.01	**−8.14 ± 2.09•10–6** ***	MDD	4.14 ± 11.35•10–3	1.75 ± 2.79•10–6
			BD	−2.15 ± 12.26•10–3	2.66 ± 3.24•10–6
Sagittal stratum R	0.45 ± 0.01	**−1.14 ± 21.79•10–6** ***	MDD	6.14 ± 13.06•10–3	1.25 ± 3.20•10–6
			BD	−8.34 ± 141.1•10–4	4.62 ± 3.73•10–6
External capsule L	0.29 ± 0.01	−4.77 ± 1.50•10–6**	MDD	7.91 ± 9.02•10–3	−1.50 ± 2.21•10–6
			BD	2.27 ± 9.74•10–3	−2.83 ± 2.57•10–6
External capsule R	0.35 ± 0.01	**−5.61 ± 1.63•10–6** ***	MDD	−4.51 ± 9.80•10–3	−1.37 ± 2.40•10–6
			BD	−6.79 ± 10.58•10–3	−1.96 ± 2.79•10–6
Middle cerebellar peduncle L	0.49 ± 0.01	−3.07 ± 1.36•10–6*	MDD	−1.17 ± 8.15•10–3	1.80 ± 2.00•10–6
			BD	−8.57 ± 8.81•10–3	1.07 ± 2.33•10–6
Middle cerebellar peduncle R	0.51 ± 0.01	−3.03 ± 1.30•10–6*	MDD	−3.01 ± 7.84•10–3	−6.59 ± 19.24•10–7
			BD	−7.12 ± 8.47•10–3	−5.75 ± 22.38•10–7
Fornix (column and body of fornix) L	0.25 ± 0.01	**−2.92 ± 0.38•10–5** ***	MDD	−2.49 ± 2.27•10–2	1.04 ± 0.56•10–5
			BD	**−8.41 ± 2.45•10–2** ***	1.66 ± 0.64•10 − 5*
Fornix (column and body of fornix) R	0.32 ± 0.01	**−3.52 ± 0.55•10–5** ***	MDD	8.13 ± 32.87•10–3	5.94 ± 8.06•10–6
			BD	−7.16 ± 3.55•10–2*	9.72 ± 9.37•10 − 6
Genu of corpus callosum L	0.64 ± 0.01	**−1.72 ± 0.27•10–5** ***	MDD	−3.95 ± 16.00•10–3	2.13 ± 3.92•10–6
			BD	−3.24 ± 1.73•10–2	2.81 ± 4.56•10–6
Genu of corpus callosum R	0.65 ± 0.01	**−1.71 ± 0.29•10–5** ***	MDD	1.94 ± 17.21•10–3	1.59 ± 4.22•10–6
			BD	−3.66 ± 1.86•10–2*	4.25 ± 4.91•10 − 6
Body of corpus callosum L	0.58 ± 0.01	**−2.07 ± 0.30•10–5** ***	MDD	−1.74 ± 1.81•10–2	6.10 ± 4.44•10–6
			BD	**−6.53 ± 1.96•10–2** ***	8.27 ± 5.17•10 − 6
Body of corpus callosum R	0.54 ± 0.02	**−1.64 ± 0.33•10–5** ***	MDD	−1.57 ± 2.01•10–2	4.82 ± 4.93•10–6
			BD	−7.07 ± 2.17•10–2**	7.94 ± 5.74•10 − 6
Splenium of corpus callosum R	0.70 ± 0.01	**−9.49 ± 2.46•10–6** ***	MDD	2.52 ± 14.72•10–3	2.08 ± 3.61•10–6
			BD	−3.37 ± 1.59•10–2*	8.66 ± 4.20•10 − 6*
Retrolenticular part of internal capsule L	0.43 ± 0.01	−3.38 ± 1.32•10–6*	MDD	1.62 ± 0.79•10–2*	−1.52 ± 1.95•10 − 6
			BD	−1.80 ± 8.58•10–3	1.81 ± 2.27•10–6
Retrolenticular part of internal capsule R	0.44 ± 0.01	−3.98 ± 1.64•10–6*	MDD	5.35 ± 9.83•10–3	−4.13 ± 24.12•10–7
			BD	−1.08 ± 1.06•10–3	1.34 ± 2.80•10–6

* *P* < 0.05.

** *P* < 0.01.

*** *P* < 0.001.

Significant *P*‐values are set in bold letters after multiple comparison correction, which required *P* < 0.001.

BD, bipolar disorder; FA, fractional anisotropy; HC, healthy control; L, left; MDD, major depressive disorder; R, right.

### Effects of duration, group, and Duration × Group interaction on the average FA and FA in the 54 tracts of MDD and BD patients

Beta coefficients and significance for the fixed effects of duration of illness, group, and Duration × Group interaction for the average FA and the FA in all the tracts are summarized in Table S3. There was a significant main effect of group in the bilateral splenium of the corpus callosum. There was no significant main effect of duration of illness or Duration × Group interaction.

### Post‐hoc comparison of FA value in white matter regions when main effects of diagnosis were detected between the groups

Post‐hoc group comparisons using Bonferroni's test indicated that FA in these white matter tracts were significantly lower in BD patients compared to MDD patients and HC (the left column and body of fornix: BD < MDD, *P* < 0.05, BD < HC, *P* < 0.05; the left body of the corpus callosum: BD < MDD, *P* < 0.01, BD < HC, *P* < 0.001; Table 4).

**Table 4:** FA values for MDD patients, BD patients, and HC and the results of post‐hoc comparison using Bonferroni's test in white matter regions where main effects of diagnosis were detected

	FA value			*P*		
	MDD	BD	HC	MDD vs BD	BD vs HC	MDD vs HC
Fornix (column and body of fornix) L	0.20 ± 0.06	0.18 ± 0.05	0.20 ± 0.07	0.026	0.031	1.000
Body of the corpus callosum L	0.53 ± 0.05	0.50 ± 0.05	0.53 ± 0.05	0.001	<0.001	1.000

BD, bipolar disorder; FA, fractional anisotropy; HC, healthy controls; L, left; MDD, major depressive disorder; R, right.

## Discussion

To the best of the authors’ knowledge, this study is the first to directly compare age‐associated changes in white matter integrity among MDD patients, BD patients, and HC. The main finding of this study was that there was significant FA reduction in the left column and body of the fornix and the left body of the corpus callosum in BD patients after controlling for age, whereas there was no Age × Group or Age^2^ × Group interaction in either disorder. The main group effect was significant after including mood states, sex, and medication load as covariates. In contrast, there was neither a significant main effect of illness duration nor a Duration × Group interaction. These results suggest that white matter abnormalities in BD patients were already present at a younger age, rather than developing progressively with age.

White matter abnormalities in children and adolescents with BD have been reported in previous DTI studies.^31^ These studies support our finding that a significant FA reduction was already present at a younger age in BD patients compared to HC. The findings of this study also indicated a significant FA reduction in BD compared to MDD regardless of age. White matter abnormalities in children and adolescents with MDD have also been reported in specific DTI studies.^5^ However, our results show more significant changes in white matter in BD compared to MDD through the life span. If this were not the case, age‐related progressive mechanisms might have had a reduced effect on disease‐specific differences in white matter abnormalities among the three groups. This result was not predicted, because other studies have reported an accelerated age‐related decline in white matter in BD.^27^, ^28^ For example, Dev *et al*.^27^ analyzed 30–69‐year‐old BD patients and reported an accelerated age‐related decline in the uncinate fasciculus and hippocampal portion of cingulum compared to HC. Toteja *et al*.^28^ analyzed 9–61‐year‐old BD patients and showed an accelerated age‐related decline in the genu of the corpus callosum compared to HC. However, it remains possible that differences such as the number of participants, the age range of participants, the targeted white matter tracts, and the image analysis method between the current and previous studies contributed to different results. Our results indicated neither a main effect of the group nor an interaction in any white matter tract of MDD patients. The study by the ENIGMA MDD working group, which is the largest MDD study to date, detected a Group × Age interaction in the genu of the corpus callosum, the body of the corpus callosum, the fornix/stria terminalis, and the sagittal stratum.^25^ We evaluated these regions to test a priori hypotheses about the uncorrected threshold (*P* < 0.05). However, no significant interaction was detected. This inconsistency might be caused by underpowered statistics, mismatches in participants’ characteristics, or different methods of DTI data analysis. Appropriate sample sizes and controls for these variables should be included in future studies.

We also found significant main effects of the group in the white matter tracts of the corpus callosum and the fornix. The large‐scale ENIGMA‐DTI workgroup's research showed significant deficits in the fornix tract in BD but not in MDD, which was consistent with our findings.^15^ These two tracts are commissural fibers that connect the left and right cerebral hemispheres, which generally show earlier maturation than association fibers and projection fibers. In particular, the fornix reaches its peak FA before the age of 20 years and has been reported as one of the tracts exhibiting the earliest maturation of whole white matter tracts during infancy and childhood.^19^, ^32^ Therefore, our results suggest that early maturing fibers are more easily damaged in individuals with BD compared to fibers that take a longer time to mature. The corpus callosum is the largest white matter structure of the human brain. It connects the cerebral hemispheres and provides interhemispheric integration and the transfer of information.^19^, ^33^ The fornix is a commissural and projection fiber located on the medial aspect of the cerebral hemisphere and constitutes the main efferent system of the hippocampus. Components of the fornix lie to either side of the midsagittal plane and connect across that plane.^34^, ^35^ Abnormal white matter integrities in these commissural fibers might result in interhemispheric disconnection. Shobe has described the importance of collaborative activities of the cerebral hemispheres via the commissural fibers in her model of emotional processing.^36^ She proposes that the right hemisphere directly mediates the identification and comprehension of positive and negative emotional stimuli, and that this emotional information is shared with the left hemisphere via the corpus callosum. Therefore, the interhemispheric disconnection in BD could disturb the exchange of emotional information and result in emotional dysregulation.^37^ The corpus callosum and fornix also contribute to working memory, problem‐solving,^38^ and memory.^35^, ^39^ MDD and BD patients are known to show cognitive impairment in different domains. Moreover, it has been reported that specific aspects of domains, such as attention and memory, are more severely impaired in BD patients.^40^, ^41^, ^42^ Meyer *et al*. also reported that BD in childhood results in increased dysfunction of executive functions compared to MDD.^43^ It is suggested that our results provide a neural basis for understanding the difference in clinical features between MDD and BD reported in the studies discussed above.

Several limitations of this study should be considered when interpreting the findings. First, our statistical design might have been too underpowered to detect the secondary (Age × Group) and tertiary (Age^2^ × Group) effects in the three groups and in multiple areas. Second, the study focused on the relation between age and white matter integrity using a cross‐sectional design. Therefore, its findings must be validated by a longitudinal study evaluating the time‐dependent changes of white matter structures. Third, patients in depressed and euthymic states were included in the study, even though we controlled for the effects of the mood during data analysis by treating the mood state as a covariate. The contribution of the mood state to white matter structure is unclear because only a few studies have undertaken group comparisons of the mood state.^4^ Nevertheless, specific studies have reported possible state‐dependent microstructural white matter change in MDD and BD.^44^, ^45^ Therefore, it would be desirable to match the mood state of the participants in future studies. Fourth, the patient groups were not matched for several clinical variables, such as their medication status, the illness severity, the number of depressive episodes, and the illness duration. We found no effect of the illness duration in our statistical analysis, which might indicate that the burden of disease contributes less to white matter changes. However, in contrast to our findings, white matter alterations associated with severity and duration of illness have been reported in several studies.^46^, ^47^, ^48^, ^49^, ^50^ Moreover, reviews and meta‐analytical studies have not reported strong evidence on the contribution of medication to changes of white matter integrity.^4^, ^5^ In contrast, other studies have reported that mood stabilizer and antidepressants might normalize the changes of FA.^45^, ^51^ Therefore, the possibility that differences in FA between the groups were a mere reflection of extraneous variables cannot be excluded. Fifth, it is possible that MDD patients convert to BD over time. We confirmed in advance that participants with MDD had no family history of BD. Sixth, our atlas‐based fiber‐tracking method had limited fiber resolution, including the crossing fiber regions, and limited accuracy in determining the origin and destination of targeted bundles.^29^ Finally, we used the Bonferroni correction method, which is a conservative test. Therefore, the possible underestimation of significance levels must be taken into consideration.

In conclusion, this study directly compared age‐associated changes in white matter integrity among MDD patients, BD patients, and HC, and found that white matter abnormalities could be detected at an earlier age in BD patients, especially in commissural fibers. Our results suggest that abnormal white matter integrity in BD might occur at a younger age rather than develop progressively with age. Moreover, it is possible that interhemispheric disconnection at an early stage of the illness could form the neural basis of differences in clinical features between MDD and BD.

## Author contributions

Y.M. designed the study, and wrote the initial draft of the manuscript. S.O. and T.K. contributed to processing and analysis of MRI data, and assisted in the preparation of the manuscript. All other authors have contributed to data collection and interpretation, and critically reviewed the manuscript. All authors approved the final version of the manuscript, and agree to be accountable for all aspects of the work in ensuring that questions related to the accuracy or integrity of any part of the work are appropriately investigated and resolved.

## Disclosure statement

The author declares no conflict of interest.

## Supporting information


**Table S1** Beta coefficients and significance for effect of age, group, and Age × Group interaction for the average fractional anisotropy (FA) and the FA values in 54 white matter tracts based on linear age effect model for major depressive disorder patients, bipolar disorder patients, and healthy controls.

**Table S2** Beta coefficients and significance for effect of age^2^, group, and Age^2^ × Group interaction for the average fractional anisotropy (FA) and the FA values in 54 white matter tracts based on quadratic age effect model for major depressive disorder patients, bipolar disorder patients, and healthy controls.

**Table S3** Beta coefficients and significance for effect of duration of illness, group, and Duration × Group interaction for the average fractional anisotropy (FA) and the FA values in white matter tracts in 54 white matter tracts based on linear duration of illness effect model for major depressive disorder patients and bipolar disorder patients.
